# Physical, chemical, and optical data of SnS layers and light switching frequency dependent photoresponses

**DOI:** 10.1016/j.dib.2017.07.056

**Published:** 2017-07-26

**Authors:** Malkeshkumar Patel, Hong-Sik Kim, Joondong Kim

**Affiliations:** Photoelectric and Energy Device Applications Lab (PEDAL) and Department of Electrical Engineering, Incheon National University, 119 Academy Rd. Yeonsu, Incheon 406772, South Korea

**Keywords:** SnS, Vertical growth, Photodetectors, 2D materials, Waferscale

## Abstract

In this data article, vertically grown SnS layers were investigated. The growth processes of vertical SnS layers were discussed in our article [1]. This data article provides the chemical analysis using the XPS measurements for the SnS sample grown on a Si wafer. Deposition time varying SnS morphology changes were observed by FESEM. The cross-sectional images were achieved to monitor the SnS layer thickness. Refractive index of the grown SnS film was calculated using the reflectance data. A self-operating photoelectric was realized with structuring of SnS layers on the n-type Si wafer. Transient photoresponses were achieved by tuning the switching frequencies.

**Specifications Table**

**Table:** 

Subject area	*Physics, Electrical Engineering*
More specific subject area	Materials Science, Photodetector
Type of data	*Figures, Images*
How data was acquired	Field emission scanning electron microscope (FESEM, JOEL, JSM_7800 F)
	X-ray photoelectron spectroscopy (XPS, PHI 5000 VersaProbe ll)
	UV-visible diffused reflectance photo spectrometer (Simadzu, UV-2600)
	Potentiostat/Galvanostat (ZIVE SP1, WonA Tech, Korea)
Data format	*Analyzed*
Experimental factors	*FESEM (Working distance 10 mm, 15 kV, specimen without conducting coating)*
	*XPS (samples prepared on the Si wafer, scan time 30 min, full scan)*
	*Light source (wavelength 850 nm, various frequency, square wave)*
	*Current-time (Chronoamperometry, Bias 0 V to -1.5 V, data interval 40 μs)*
Experimental features	*Vertically grown SnS layers*
Data source location	*Incheon National University, Incheon 22012, Korea*
Data accessibility	*The data are with this article*

**Value of the data**

• *XPS data of the grown SnS samples are prepared for the SnS chemical information.*
• *FESEM images provided the surface morphologies of the vertically grown SnS layers with deposition time variation.*
• *Refractive index and reflectance data of SnS sample could be useful in the designing of photoelectric devices.*
• *The vertical SnS layers were applied for a high-performing photodetector.*

## 1 Data

**Fig. 1:**
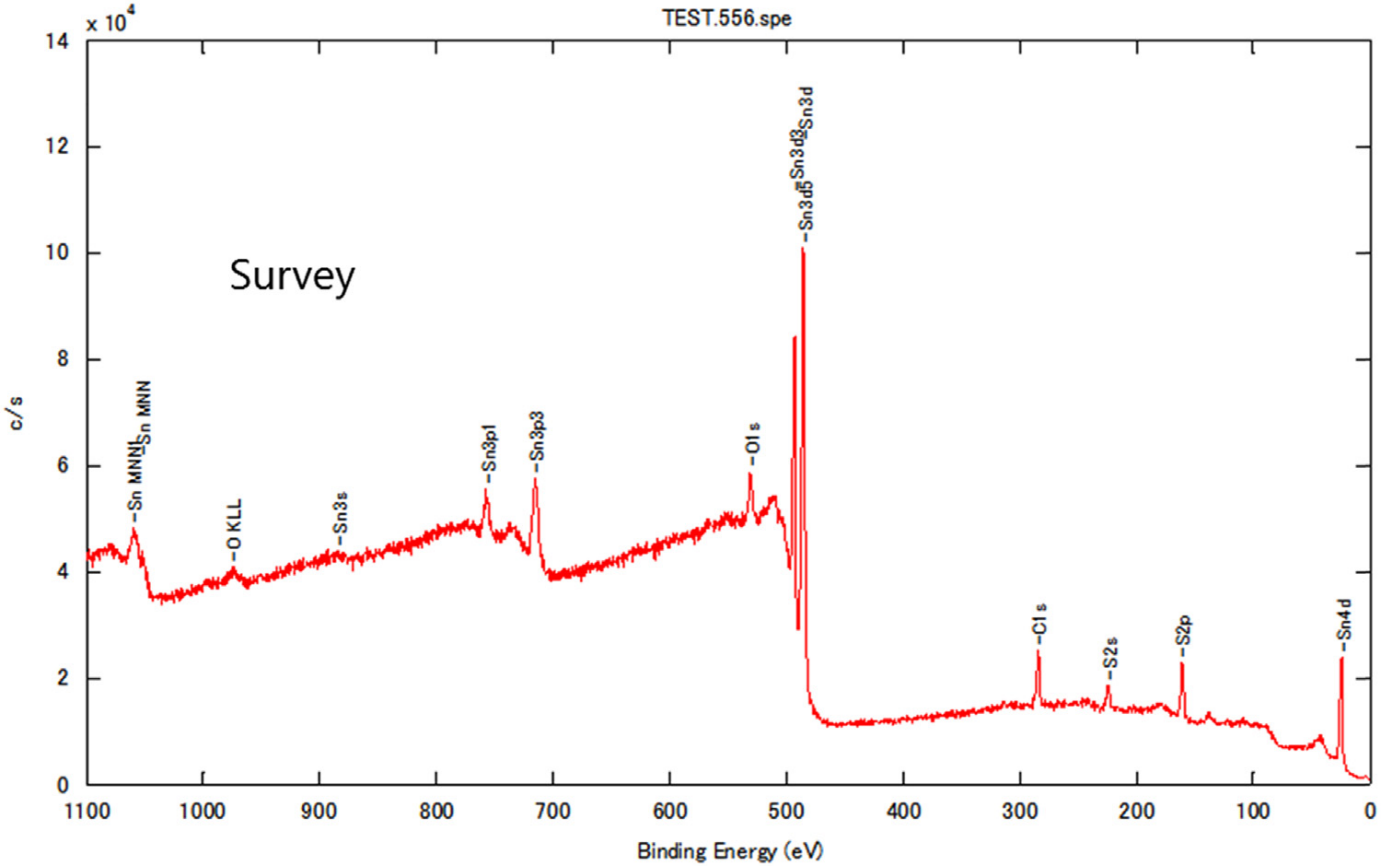
XPS survey spectra of vertically grown SnS sample on a Si wafer.

**Fig. 2:**
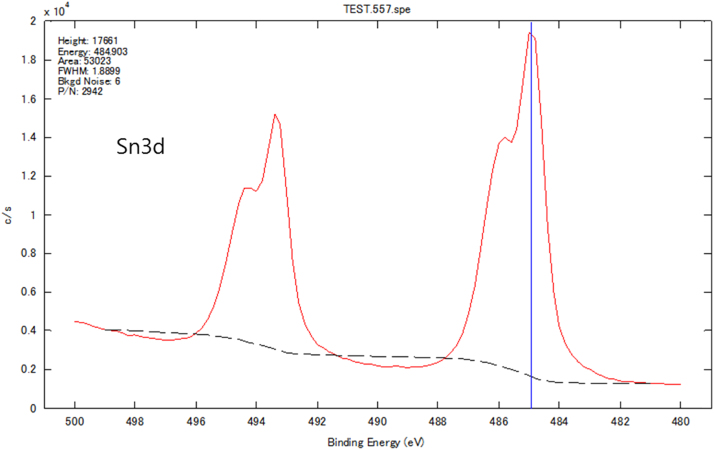
XPS Sn3d spectra of vertically grown SnS sample on a Si wafer.

**Fig. 3:**
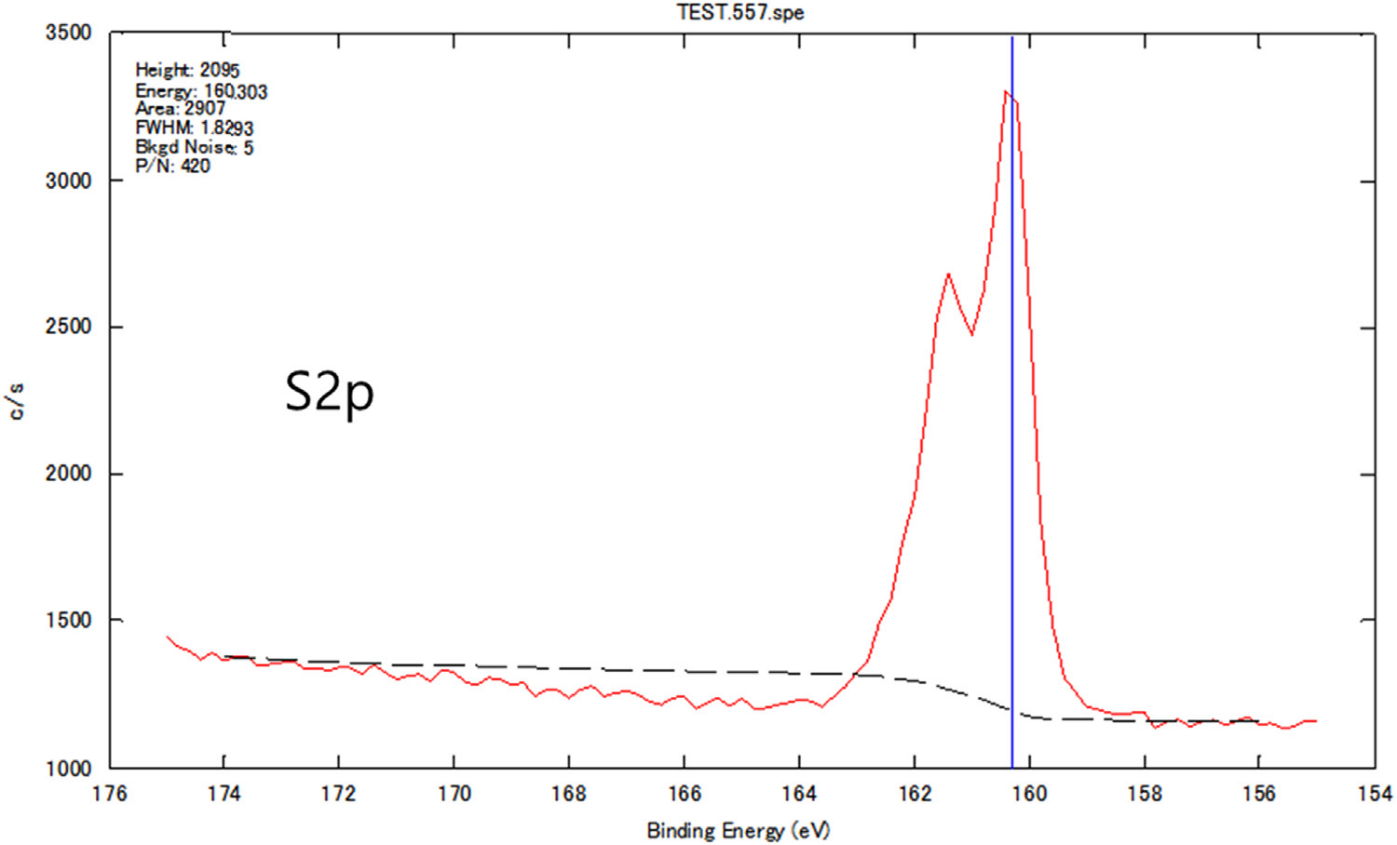
XPS S2p spectra of vertically grown SnS sample on a Si wafer.

**Fig. 4:**
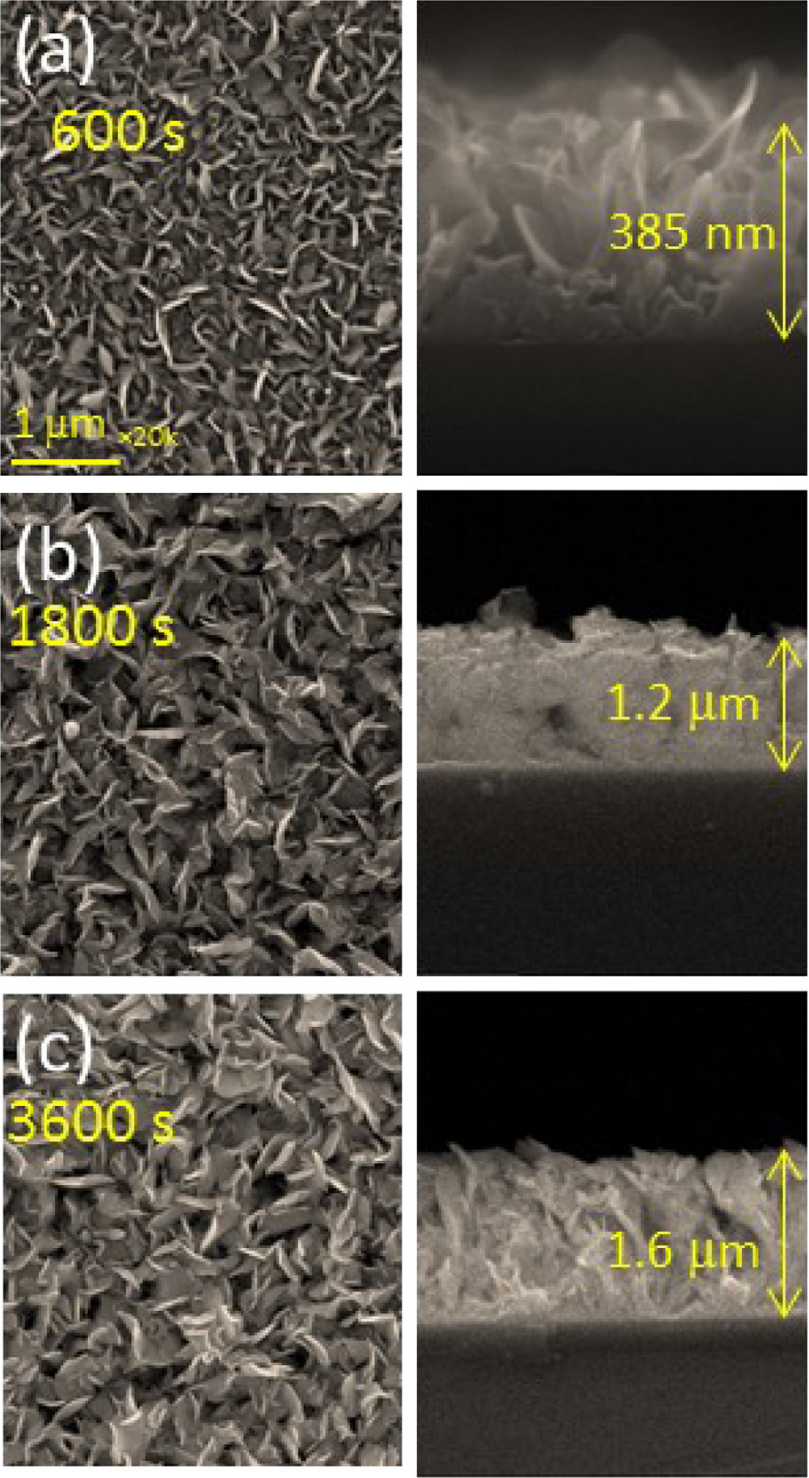
FESEM images of surface morphology and cross-section of the vertically grown SnS layers on a Si wafer for various deposition time (a) 600 s, (b) 1800s, and (c) 3600 s.

**Fig. 5:**
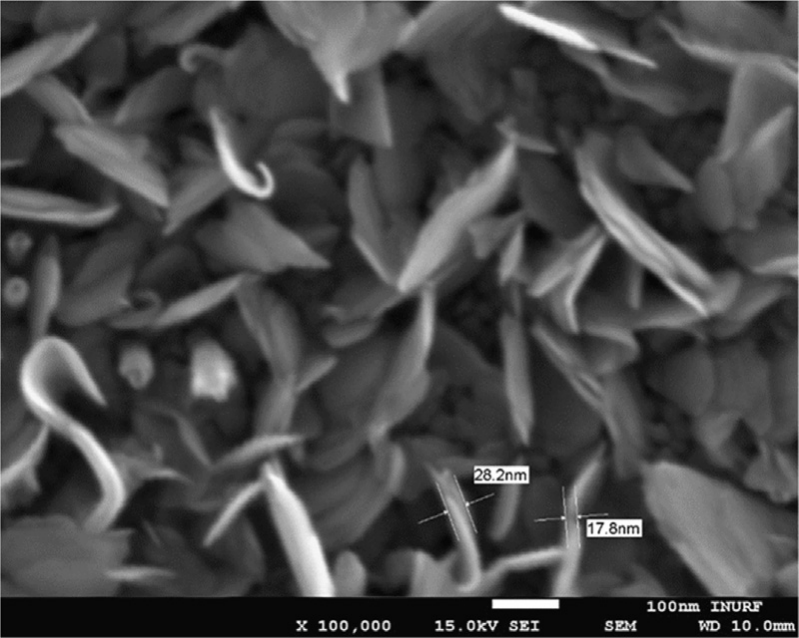
FESEM image of surface morphology to estimate the SnS layer thickness.

**Fig. 6:**
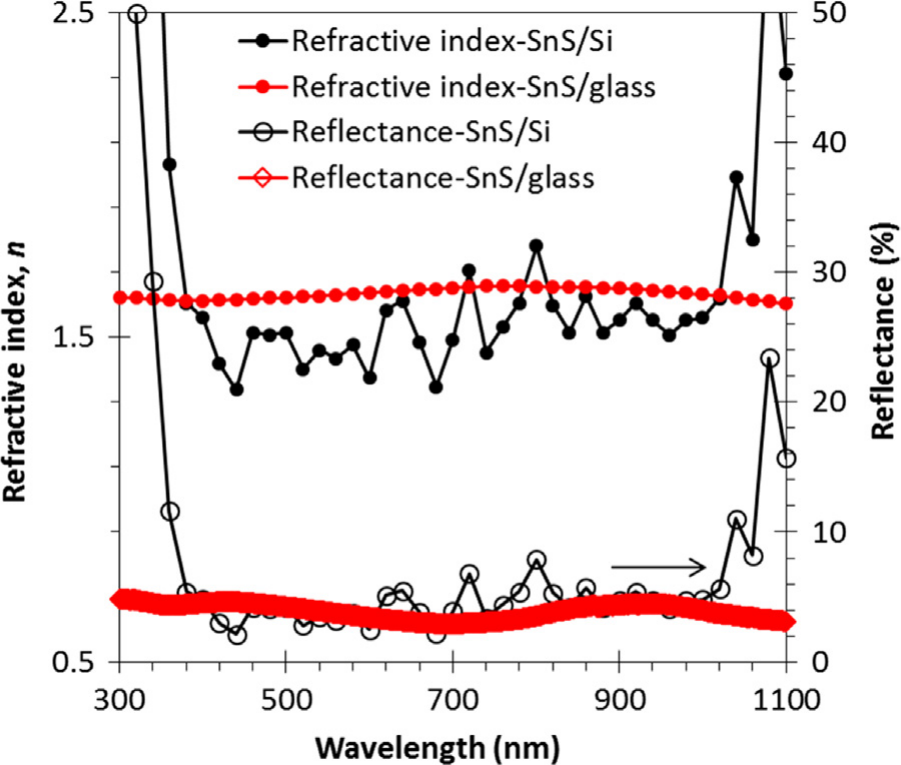
Refractive index of the SnS samples grown on the glass and Si wafer. These data were calculated using the measured reflectance data as shown on the secondary axis.

**Fig. 7:**
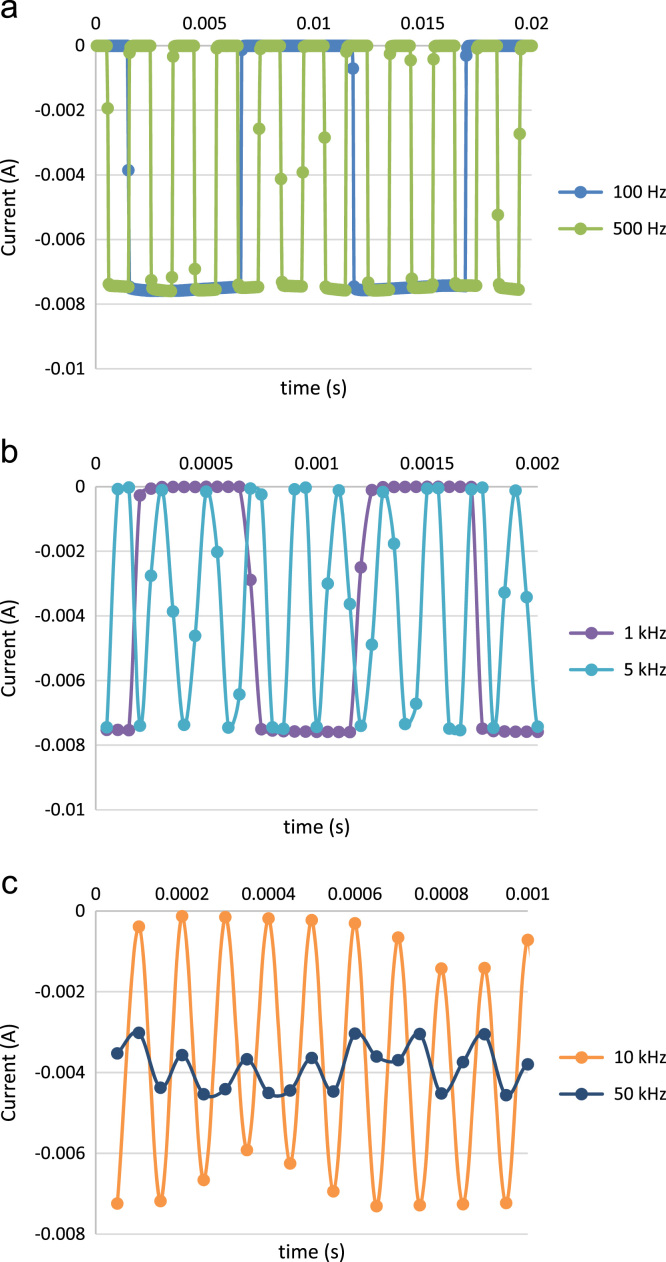
Effect of switching frequency of light source on the current-time characteristics of the SnS/n-Si photoelectric device operated at zero bias. (a) 100–500 Hz, (b) 1–5 kHz, and (c) 10–50 kHz.

Chemical analyses of the prepared SnS thin film based upon XPS analysis are provided in Fig. 1, Fig. 2, Fig. 3. XPS survey spectra including the Sn3d and the S2p spectra confirm the chemical states corresponding to the Sn^+2^ and S^-2^ states. The thickness-dependent surface morphology and cross-sectional FESEM images of vertical layers of SnS stacked on Si wafer are shown in Fig. 4. Cross-sectional images of SnS samples demonstrated the length extension of the vertical SnS layer for a wafer-scale application [1]. This vertical SnS could be one of emerging 2D materials, such as HfS_2_, [2] MoS_2_, [3] MoSe_2_ and WSe_2_, [4] ReS_2_, [5] and SnS_2_, [6]. The thickness of multilayers of SnS was estimated by using the FESEM image, as shown in Fig. 5. Refractive index of the grown SnS films was calculated using the reflectance data. Both reflectance and refractive index data of SnS film are shown in Fig. 6. Photoresponse data of SnS/n-Si device and light switching frequency dependent are shown in Fig. 7. The light source of wavelength 850 nm was used to acquire these data.

## 2 Experimental design, materials and methods

### 2.1 SnS sample preparation and device making

Vertically oriented SnS layers [1] were achieved by using the reactive RF sputtering from a SnS_2_ target material. The reactive process at 300 °C of substrate temperature induced the phase structural transition and sulfur dissociation in SnS_2_ deposits. In order to fabricate the photodetector, the vertical SnS layers were formed on an n-type Si. A transparent conductor of ITO was capped onto the SnS layers to sever a front contact. To make a rear contact, Al electrode was deposited on the back of the n-Si substrate.

### 2.2 Sample characterizations

The chemical analysis of vertically grown SnS sample as shown in Fig. 1, Fig. 2, Fig. 3 was obtained using the X-ray photoelectron spectroscopy (XPS, PHI 5000 VersaProbe ll). The cross-sectional, surface morphology of vertically grown SnS samples were captured by using a field emission scanning electron microscope (FESEM, JEOL, JSM_7800F) (Fig. 4, Fig. 5). Reflectance data of the SnS sample was recorded between the wavelength ranges from 100 nm to 300 nm (Fig. 6). Diffused integrating sphere was used to mount the SnS sample. Near infrared photodetection properties of the fabricated SnS device was studied by tuning the light illumination frequencies. Chronoamperometry technique was performed to study the photocurrent of the device. The acquired data are shown in the Fig. 7.
