# Supplementary material for: Physical, chemical, and optical data of SnS layers and light switching frequency dependent photoresponses
Source: Data Brief. 2017 Jul 26;14:206–12. doi: 10.1016/j.dib.2017.07.056 (PMC5545874; doi:10.1016/j.dib.2017.07.056)
Supplement: Supplementary file 1 — Supplementary material [file mmc1.docx]

***Conflicts of Interest Statement***

Re: DIB-D-17-00628

Title: Physical, chemical, and optical data of SnS Layers and Light Switching Frequency Dependent Photoresponses

We declare that this manuscript is original, has not been reported before, and is not currently being considered elsewhere. We also confirm that there is no known conflict of interest regarding this manuscript and its publication. The manuscript has been approved by all named authors.

Sincerely yours,


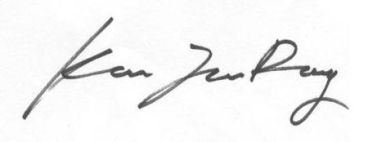


Joondong Kim

Joondong Kim, Ph.D./Professor

Department of Electrical Engineering,

Incheon National University

E-mail: joonkim@ incheon.ac.kr

Phone: +82-32-835-8770; fax: +82-32-835-0773
